# Learning from health system actor and caregiver experiences in Ghana and Nepal to strengthen growth monitoring and promotion

**DOI:** 10.1371/journal.pone.0282807

**Published:** 2023-03-09

**Authors:** Akriti Singh, Kelsey A. Torres, Nashna Maharjan, Jyoti Shrestha, Faith Agbozo, Abdulai Abubakari, Lutuf Abdul-Rahman, Altrena Mukuria-Ashe

**Affiliations:** 1 USAID Advancing Nutrition, Helen Keller International, New York, New York, United States of America; 2 USAID Advancing Nutrition, JSI Research & Training Institute, Inc., Arlington, Virginia, United States of America; 3 Mother and Infant Research Activities, Kathmandu, Bagmati Province, Nepal; 4 Department of Family and Community Health, University of Health and Allied Sciences, Hohoe, Volta Region, Ghana; 5 Department of Global and International Health, University for Development Studies, Tamale, Northern Region, Ghana; 6 USAID Ghana, Accra, Greater Accra Region, Ghana; 7 USAID Advancing Nutrition, Save the Children, Washington, DC, United States of America; Menzies School of Health Research, AUSTRALIA

## Abstract

**Background:**

Globally, growth monitoring and promotion (GMP) of infants and young children is a fundamental component of routine preventive child health care; however, programs have experienced varying degrees of quality and success with enduring challenges. The objective of this study was to describe implementation of GMP (growth monitoring, growth promotion, data use, and implementation challenges) in two countries, Ghana and Nepal, to identify key actions to strengthen GMP programs.

**Methods:**

*W*e conducted semi-structured key informant interviews with national and sub-national government officials (n = 24), health workers and volunteers (n = 40), and caregivers (n = 34). We conducted direct structured observations at health facilities (n = 10) and outreach clinics (n = 10) to complement information from interviews. We coded and analyzed interview notes for themes related to GMP implementation.

**Results:**

Health workers in Ghana (e.g., community health nurses) and Nepal (e.g., auxiliary nurse midwives) had the knowledge and skills to assess and analyze growth based on weight measurement. However, health workers in Ghana centered growth promotion on the growth trend (weight-for-age over time), whereas health workers in Nepal based growth promotion on measurement from one point in time to determine whether a child was underweight. Overlapping challenges included health worker time and workload. Both countries tracked growth-monitoring data systematically; however, there was variation in growth monitoring data use.

**Conclusion:**

This study shows that GMP programs may not always focus on the growth trend for early detection of growth faltering and preventive actions. Several factors contribute to this deviation from the intended goal of GMP. To overcome them, countries need to invest in both service delivery (e.g., decision-making algorithm) and demand generation efforts (e.g., integrate with responsive care and early learning).

## Introduction

**Fig 1:**
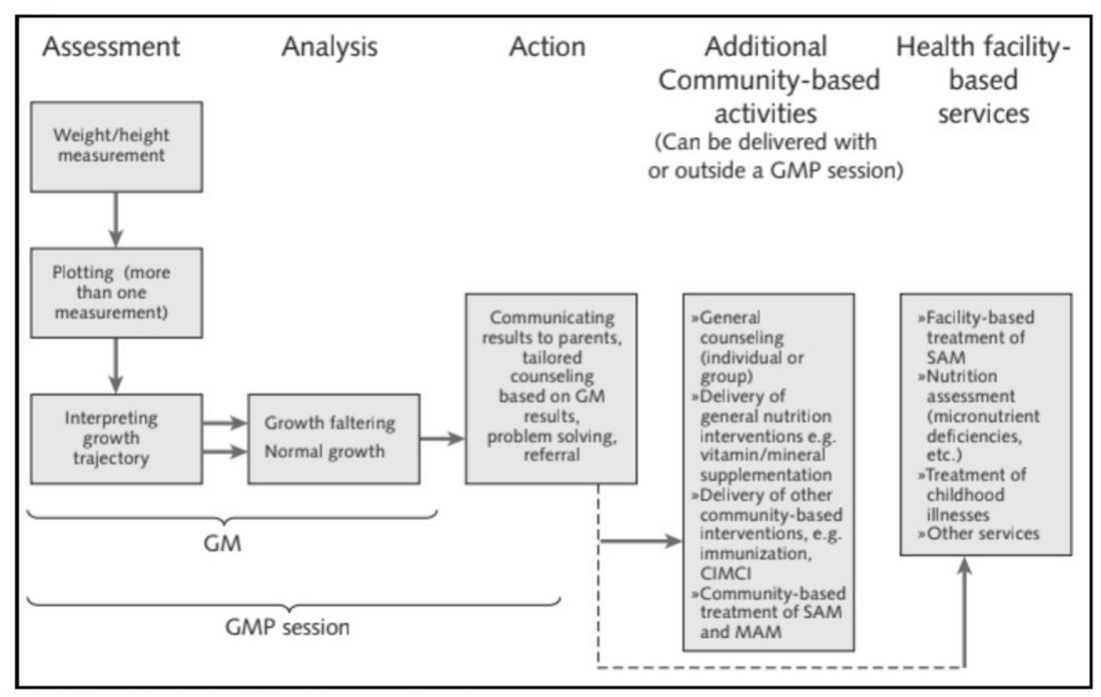
GMP process. Adapted from: Mangasaryan, Arabi, and Schultink, 2011, P. 47, CIMCI: community-based integrated management of childhood illness | SAM: severe acute malnutrition | MAM: moderate acute malnutrition.

Globally, 178 countries use growth monitoring and promotion (GMP) of infants and young children as a fundamental component of routine preventive child health care [1]. Growth monitoring, the first step in GMP, consists of assessing (monthly weight or height measurement, plotting, and interpreting the growth trajectory) and analyzing the trend for normal or faltering growth [2]. The second component of GMP, growth promotion, involves linking the growth trend from growth monitoring with actions (i.e., communicating results to caregivers, tailoring counseling based on the growth trend, problem solving, and referral) for creating caregiver awareness about child growth, improving care practices, and increasing demand for other services as needed (Fig 1) [2,3].

When delivered with quality in a functional health system, GMP is associated with significant reductions in malnutrition and mortality [4]. However, GMP is implemented with varying degrees of quality, so evidence for the effectiveness of GMP is difficult to generate, insufficient, and inconsistent [1,2,4,5]. Transitioning from successful and high fidelity small-scale programs to nationwide coverage with quality has been particularly challenging [6]. Rather than assessing the growth trend, which enables health workers to detect early growth faltering and tailor counseling, some programs focus more on a single point in time, screening for underweight [1,2,5,7]. In these contexts, children who are growth faltering but not yet underweight are not detected, so the goal of reaching children who are at risk of malnutrition early with preventive services is not achieved. Many countries conducting growth monitoring fail to use growth data to guide decision-making and action, especially when scaling up, which undermines the effectiveness of the program [2,8]. Furthermore, there is a missed opportunity to leverage GMP for monitoring and supporting children’s physical, language, cognitive, and socio-emotional development, despite strong evidence that integrated services can help improve development outcomes [9,10].

In 2018, a global convening of thought leaders, program implementers, and researchers reviewed evidence, discussed challenges, and proposed opportunities to refresh GMP [1]. Participants articulated a compelling need for GMP programs and issued a call to think through the role of GMP in the global push for integrated child growth and development approaches encouraged by the Sustainable Development Goals [1]. The objective of this study was to inform this global conversation by describing implementation of GMP in two countries, Ghana and Nepal. Specifically, we aimed to answer the following questions: 1) How is growth monitoring implemented? 2) How is growth promotion implemented? 3) How are growth trends tracked and used for program implementation? 4) What are the challenges to quality GMP from the perspectives of caregivers, health workers, and government officials?

## Methods

### Study design and case selection

We chose a case study design due to its strength for in-depth exploration of how a program (in this case, GMP) is implemented within its natural context from multiple perspectives [11,12]. We used a multiple case approach (with the country as the unit of analysis) to describe implementation in two different contexts [11]. We selected Ghana and Nepal because both countries have implemented GMP for decades and provide a learning opportunity in two geographic regions (Africa and Asia). Key elements of the study design were similar in both countries: the type of participants, sampling process, and data collection guides, which we tailored to each country.

### Study context

#### Ghana

GMP has been a core child health and nutrition service delivery platform in Ghana since the 1970s [13]. The Ghana Health Service (GHS) aims to reach all children under five years of age with GMP services through monthly contacts from birth to one year of age, quarterly contacts for children one to two years of age, and bi-annual contacts for children from three to five years of age. Trained health workers, mostly community health nurses (CHN), deliver GMP services through child welfare clinics at facilities, community-based health planning and services compounds, and mobile outreach clinics with support from community health volunteers [14].

#### Nepal

In Nepal, the Ministry of Health and Population (MoHP) has implemented GMP for over 20 years as a core child health and nutrition service [15]. The MoHP aims for monthly contacts for children from birth to two years of age [16]. With support from female community health volunteers (FCHVs), trained auxiliary health workers and auxiliary nurse midwives deliver GMP through health facilities and monthly primary health care outreach clinics (PHC ORCs) where they offer additional primary health care services, such as antenatal care, family planning, health education, and counseling. The Expanded Program on Immunization clinics may also be held on the same day (and location) as PHC ORCs.

### Data collection

In Ghana, we purposively selected two U.S. Agency for International Development (USAID) Ghana priority districts in northern Ghana for diversity of regions and variation in nutritional status, GMP attendance, and accessibility for researchers. Knowledge of stakeholders from GHS and USAID Ghana and GHS data informed selection of East Mamprusi Municipal Assembly in the North East Region and Garu District in the Upper East Region. The team collected data between November 2020 and January 2021. Prior to the interviews and observations, the investigators virtually oriented the data collection team. The team pretested the interview and observation guides in Tamale Metropolitan District in the Northern Region.

In Nepal, we purposively selected three districts based on stakeholder knowledge (in consultation with the Chief of Nutrition Section in the Family Welfare Division at the MoHP, USAID Nepal, and the USAID-funded Suaahara II project) and data from the Ministry of Health and Population. We selected these districts to represent diversity in agro-ecological zones (Kailai in the Terai region, Jajarkot in the hills, and Sindhupalchowk in the mountains); provinces; and average annual frequency of GMP visits for children under 24 months of age (Jajarkot: 2.1; Sindhupalchowk: 3.1; Kailali: 4.2) [17]. The team collected data between April 2021 and July 2021. Prior to the interviews and observations, the investigators virtually oriented the data collection team. The team pretested the interview and observation guides in Lalitpur District in Bagmati Province.

#### Desk review

We conducted a desk review of policies, strategies, protocols, program reports, and peer-reviewed literature to understand implementation of GMP, challenges faced, data reporting systems, and use of data for decision-making in both countries. This desk review informed the design of our data collection guides. The data collection guides examined whether health workers implemented GMP in alignment with the Assess-Analyze-Act framework for GMP [2].

#### Interviews

We conducted semi-structured key informant interviews with health system actors (national and sub-national government officials, health workers, and volunteers) and caregivers (Table 1) in order to gather perspectives from actors who play different roles in GMP (e.g., oversee, implement, or attend). In Nepal, we also interviewed FCHVs since they play a critical role in community health and nutrition services. We aimed to conduct enough interviews in each respondent category within each country to reach data saturation. Our sample sizes were in line with studies that have assessed how many interviews need to be done within a relatively homogenous population to reach saturation [18].

**Table 1:** Data collection methods, types of participants, and observation sites.

Method	Type	Ghana				Nepal					Total
		National	East Mamprusi	Garu	Sub-Total	National	Jajarkot	Sindhupalchowk	Kailali	Sub-Total	
Semi-structured interviews	Development partner	5	0	0	5	5	0	0	0	5	10
	Government official	2	6	4	12	3	3	3	3	12	24
	Health worker	N/A	8	8	16	N/A	4	4	4	12	28
	Female Community Health Volunteer (FCHV)	N/A	N/A	N/A	N/A	N/A	4	4	4	12	12
	Caregiver	N/A	10	6	16	N/A	6	6	6	18	34
Observations	Health facility	N/A	1	3	4	N/A	2	2	2	6	10
	Outreach	N/A	3	1	4	N/A	2	2	2	6	10

**Note:** Sample size varies by country due to the difference in number of districts where the study was conducted. The number of observations varies by district in Ghana due to location of child welfare clinics at the time the researchers visited the districts.

We purposively selected national and sub-national government officials based on their knowledge of the GMP program. We selected a convenience sample of health facilities based on stakeholder preferences. Within the health facilities we purposively sampled health workers (e.g., auxiliary nurse midwives in Nepal, community health nurses in Ghana), FCHVs, and caregivers (i.e., mother or father of a child under the age of 2 attending GMP) based on their experience implementing GMP or receiving GMP services. We asked participating national and sub-national officials about roles, protocols, data collection and use, and priority areas for GMP. During interviews with health workers and volunteers, we asked about experiences, challenges, linkages with other programs and platforms, and recommendations for GMP. Interviews with caregivers focused on perceptions of services, linkages with other programs and platforms, and recommendations for GMP. The number of interviews were almost equally split across the districts in each country (Table 1).

In both countries, a researcher with extensive qualitative research experience led the interview while another took notes. In Ghana, researchers conducted the interviews in English, Dagbani, Kusal, Kusasi, Mampruli, or Twi. In Nepal, researchers conducted all the interviews in Nepali. The majority of interviews were audio recorded in both countries to verify notes. Prior to data analysis, we translated the notes into English.

#### Observations

In both countries, we conducted direct structured observations of GMP sessions (Table 1) to understand how health workers took, recorded, and used growth measurements at two service points: health facilities and outreach clinics. At the health facilities and their associated outreach clinics we conducted spot checks of GMP services for 1–2 caregiver-child pairs per health facility or outreach clinic [19]. The sessions were 5–40 minutes in duration (Table 4). To observe the GMP sessions, we adapted the structured observation checklist developed by the Uganda Program for Human and Holistic Development to review which growth measurements were taken, whether health workers assessed and analyzed them, and if/how they counseled caregivers (Table 4) [20]. One data collection team member completed the checklist while observing a caregiver-child pair’s GMP session.

#### Validation workshops

In each country, we convened two rounds of workshops with government officials and development partners. The first round took place pre-data collection to solicit inputs on the study design; the second round occurred post-data collection to validate findings and refine analysis in light of GMP practices and priorities.

### Data analysis

We used applied thematic analysis to identify themes in the data [21]. To do this, we developed a codebook for each country using deductive or pre-determined codes and inductive codes based on emerging themes. Using ATLAS.ti 9 software, analysts coded 10 percent of the interview notes in each country and compared their work to ensure consistent interpretation and use of the codebook. Using these insights, we discussed any needed revisions and updated the codebook along with the coded interview notes. The analysts then split the remaining interviews amongst themselves to complete the coding. We subsequently analyzed the data by thematic area for each research question (presented above) [21]. One researcher entered data from observations in each country separately in Microsoft Excel. Researchers triangulated data from the desk review, interviews, observations, and workshops to validate and refine the findings by country and then compared them.

When reporting results, we have used qualitative terminology as is appropriate for a qualitative study. However, for observation results we also provide percentages and proportions (e.g., 58 percent, 7 of 12), as they are based on a quantitative checklist. During analysis, we used detailed interview notes rather than transcripts given the applied nature of the study (to understand how a public health nutrition program was implemented) and the absence of topics that require verbatim words (e.g., linguistic analysis, cultural themes, life histories, identity) so we have not included direct quotes [22].

### Research ethics

The study was reviewed and approved by John Snow Inc. Institutional Review Board (#20–34), the Ghana Health Service Ethics Review Committee (GHS-ERC013/10/20), and the Nepal Health Research Council (177/2021 P). All participants gave written (or thumb print) consent before interviews and observations.

### Inclusivity in global research

Additional information regarding the ethical, cultural, and scientific considerations specific to inclusivity in global research is included in the Supporting Information.

## Results

### Participant characteristics

The majority of interviewed participants were health workers or caregivers (Table 1). Most health workers were female (72 percent), however, at the country level, there was more balance across sexes in Ghana (88 percent of health workers were females in Nepal, while 50 percent were females in Ghana) (Table 2). Health workers had fewer years of experience in Ghana—69 percent had less than 5 years of experience—whereas almost two-thirds of health workers in Nepal had 10 or more years of experience. FCHVs in Nepal largely contribute to this difference with an average of 16 years of experience.

**Table 2:** Characteristics of health workers interviewed.

	Ghana		Nepal			Total Number (n = 40)
	East Mamprusi (n = 8)	Garu (n = 8)	Jajarkot n = 8)	Sindhupalchowk (n = 8)	Kailali (n = 8)	
**Sex**						
Female	5 (63%)	3 (38%)	6 (75%)	8 (100%)	7 (88%)	29 (73%)
Male	3 (38%)	5 (63%)	2 (25%)	0 (0%)	1 (13%)	11 (28%)
**Years of service**						
<5 years	5 (63%)	6 (75%)	2 (25%)	1 (13%)	1 (13%)	15 (38%)
5–9 years	2 (25%)	1 (13%)	2 (25%)	2 (25%)	1 (13%)	8 (20%)
10+ years	1 (13%)	1 (13%)	4 (50%)	5 (63%)	6 (75%)	17 (43%)
**Position**						
Community health nurse (CHN)	5 (63%)	6 (75%)	N/A	N/A	N/A	11 (28%)
Community health officer (CHO)	0	1 (13%)	N/A	N/A	N/A	1 (3%)
Auxiliary nurse midwife (ANM)	N/A	N/A	2 (25%)	3 (38%)	2 (25%)	7 (18%)
Auxiliary health worker (AHW)	N/A	N/A	2 (25%)	1 (13%)	0 (0%)	3 (8%)
Female community health volunteer (FCHV)	N/A	N/A	4 (50%)	4 (50%)	4 (50%)	12 (30%)
Other*	3 (38%)	1 (13%)	0 (0%)	0 (0%)	2 (25%)	6 (15%)

*Other positions include health assistants, nutrition focal persons, and field technician disease control officers.

Caregivers in the study ranged from age 18 to 42 years, with the average age being 29 years in Ghana and 27 years in Nepal (Table 3). Despite efforts to include fathers in the caregiver sample, we only interviewed one father in Nepal because mothers most often brought children to the health facility. Among caregivers interviewed, the average age of the child brought to GMP was 12 months in Ghana and 13 months in Nepal. Children ranged in age from 6 to 23 months in Ghana and 3 to 24 months in Nepal. Average household size was smaller in Ghana (6 people) compared to Nepal (10 people).

**Table 3:** Characteristics of caregivers interviewed and the child they brought to GMP.

	Ghana		Nepal		
	East Mamprusi (n = 10)	Garu (n = 6)	Jajarkot (n = 6)	Sindhupalchowk (n = 6)	Kailali (n = 6)
**Caregiver age (years)**					
Average	26	33	26	29	28
Range	(19–34)	(24–42)	(22–33)	(20–39)	(21–35)
**Child age (months)**					
Average	12	11	13	13	14
Range	(6–23)	(6–20)	(3–21)	(4–24)	(4–21)
**Average household size, range (people)**					
Average	12	7	6	6*	7*
Range	(4–30)	(3–14)	(4–8)	(3–8)*	(5–8)*

*Numbers based on responses from 5 of 6 caregivers who were interviewed.

### Growth monitoring

During interviews, all health workers in Ghana and Nepal described assessing and analyzing weight measurements when demonstrating their knowledge of what comprises growth monitoring. However, in practice, there was variation in whether and how health workers assessed and analyzed growth. This variation likely resulted from the quality of equipment, design of the growth chart used, availability of weight measurements over time, and health workers’ training on GMP.

#### Growth assessment

All health workers in Ghana and Nepal reported measuring weight. Some health workers in Ghana and many in Nepal also mentioned measuring length/height. Few health workers in Ghana and most in Nepal noted measuring mid-upper arm circumference (MUAC). More health workers in Nepal reported measuring length and MUAC because the same health workers implement the integrated management of acute malnutrition program, which covers measuring weight, length, and MUAC to identify and track children with acute malnutrition/wasting. Several health workers in Nepal described pausing weight measurements at the peak of the coronavirus disease of 2019 (COVID-19) pandemic. The majority of health workers in Ghana described “plotting” or “charting” weight measurements, but only a few health workers in Nepal mentioned doing so. During observed sessions, all health workers in Ghana plotted weight in the Maternal and Child Health Record Book (MCHRB), or the old child welfare card, but only 50 percent (6 of 12) of health workers in Nepal plotted weight on the child health card (Table 4). Several observed child health cards in Jajarkot District of Nepal did not have the weight measurement plotted even when the health worker updated the immunization record. A caregiver in Jajarkot shared:

*They tell us the weight verbally*. *Only the immunization is recorded*. *That’s why I didn’t bring the card today*. *Weight and arm circumference is recorded in their (health post’s) register and is not recorded in the card*.

**Table 4:** GMP practices observed in Ghana and Nepal.

	Ghana			Nepal			
	Health facility (n = 4)	Outreach (n = 4)	Sub-Total (n = 8)	Health facility (n = 6)	Outreach (n = 6)	Sub-Total (n = 12)	Total (n = 20)
**Background information (health worker)**							
Cadre							
Community health nurse (CHN)	3	3	6 (75%)	N/A	N/A	N/A	6 (30%)
Community health officer (CHO)	0	1	1 (13%)	N/A	N/A	N/A	1 (5%)
Public health nurse	1	0	1 (13%)	N/A	N/A	N/A	1 (5%)
Auxiliary nurse midwife (ANM)	N/A	N/A	N/A	4	4	8 (67%)	8 (40%)
Auxiliary health worker (AHW)	N/A	N/A	N/A	2	2	4 (33%)	4 (20%)
Sex							
Female	1	4	5 (63%)	4	4	8 (67%)	13 (65%)
Male	3	0	3 (38%)	2	2	4 (33%)	7 (35%)
Highest level of education							
11 years	0	0	2 (0%)	2	0	2 (17%)	2 (10%)
Senior high school	0	1	1 (13%)	0	0	0 (0%)	1 (5%)
Intermediate school	0	0	0 (0%)	2	0	2 (17%)	2 (10%)
Certificate	3	3	6 (75%)	0	2	2 (17%)	8 (40%)
Nursing degree	1	0	1 (13%)	0	0	0 (0%)	1 (5%)
Diploma in pharmacy	0	0	0 (0%)	0	1	1 (8%)	1 (5%)
Bachelor degree	0	0	1 (0%)	2	1	3 (25%)	3 (15%)
CMA training	0	0	3 (0%)	0	1	1 (8%)	1 (5%)
CMA training + 12 years	0	0	4 (0%)	0	1	1 (8%)	1 (5%)
Years of GMP experience							
0–5	4	3	7 (88%)	1	3	4 (33%)	11 (55%)
6–10	0	0	0 (0%)	4	0	4 (33%)	4 (20%)
11+	0	1	1 (13%)	1	2	3 (25%)	4 (20%)
Any training on GMP	4	4	8 (100%)	4	2	6 (50%)	14 (70%)
**Growth promotion session**							
Sex of child							
Female	3	1	4 (50%)	2	0	2 (17%)	6 (30%)
Male	1	3	4 (50%)	4	6	10 (83%)	14 (70%)
Weight measured	4	4	8 (100%)	6	6	12 (100%)	20 (100%)
Length measured	0	0	0 (0%)	1	0	1 (8%)	1 (5%)
Measurement plotted	4	4	8 (100%)	3	3	6 (50%)	14 (70%)
Child grew in past 30 days	2	2	4 (50%)	4*	6	10 (83%)	14 (70%)
Health worker analyzed, assessed, and communicated result to caregiver	3	3	6 (75%)	6	2	8 (67%)	14 (70%)
Health worker asked about health of child	0	0	0 (0%)	4	3	7 (58%)	7 (35%)
Health worker provided counseling	3	3	6 (75%)	5	6	11 (92%)	17 (85%)
One-on-one	1	2	3 (50%)	3	3	6 (55%)	9 (53%)
Group	2	1	3 (50%)	4	2	6 (55%)	9 (53%)
Tailored counseling	1	0	1 (17%)	6	5	11 (100%)	12 (71%)
Involved caregiver in discussion	2	0	2 (33%)	5	3	8 (73%)	10 (59%)
Gave caregiver opportunity to ask questions	2	0	2 (33%)	3	2	5 (45%)	7 (41%)
Used job aids	2	0	2 (33%)	1	0	1 (9%)	3 (18%)
Praised caregiver	0	1	1 (17%)	2	1	3 (27%)	4 (24%)
Reached an agreement	1	1	2 (33%)	2	1	3 (27%)	5 (29%)
Asked caregiver to repeat agreement	0	0	0 (0%)	0	0	0 (0%)	0 (0%)
Recorded agreement	0	0	0 (0%)	1	0	1 (9%)	1 (6%)
Told caregiver when to return	3	1	4 (50%)	5	6	11 (92%)	15 (75%)
Made a referral	0	1	1 (13%)	3	3	6 (60%)	7 (35%)
Length of session (mins.)							
0–10	1	1	2 (25%)	5	6	11 (92%)	13 (65%)
11–20	2	3	5 (63%)	0	0	0 (0%)	5 (25%)
21–30	0	0	0 (0%)	1	0	1 (8%)	1 (5%)
31+	1	0	1 (13%)	0	0	0 (0%)	1 (5%)

*One record was unclear.

#### Growth analysis

Most health workers in Nepal described interpreting growth using growth charts and identified children as normal, low weight, or very low weight based on where their weight measurement from a single visit fell on the growth chart. Many in Ghana focused on the growth trend (weight-for-age increasing or decreasing over time). The growth chart in Nepal has red, yellow, and green bands, while the chart in Ghana has colored lines highlighting the standard deviations: green for 0 (median), red for 2 standard deviations above or below the median, and black for 3 standard deviations above or below the median.

Most interviewed health workers in Ghana shared that they had been trained in GMP or a component of GMP while only a little over half of the health workers in Nepal reported doing so. In both countries, health workers described their training as specific to GMP or a related program such as infant and young child feeding or integrated management of acute malnutrition/community-based management of acute malnutrition.

#### Challenges

Health workers in Nepal reported that attendance at GMP was low due to the distance to a health facility, competing priorities among caregivers, and poor anthropometric measurement recording practices among health workers. Health workers stated that caregivers often visited multiple health facilities, which prevented documenting their information in one register, thus contributing to infrequent attendance records. Health workers used data from the register to calculate monthly GMP attendance indicators. Sub-national stakeholders commented that caregivers came for GMP, but health workers often did not record the weight measurement. As one sub-national stakeholder from Sindhupalchowk District, Nepal, shared:

*At least the child comes 7 times for immunization*, *but an average GMP visits accounts [for] only 3 to 4 visits*. *At least it should be 7 visits* … *So*, *there is the issue of recording and reporting as well*.

In contrast, health workers in Ghana did not identify attendance as a challenge for GMP. This may be because health facilities in Ghana offer GMP every day of the week, but in Nepal they only offer it on a specific day of the month. Additionally, health workers in Ghana noted that providing immunization and vitamin A supplementation to children who attended GMP improved attendance in the early years of the child’s life. However, sub-national stakeholders in Nepal shared that offering GMP and immunization on the same day contributed to low GMP attendance, as caregivers only brought children for GMP when they were due for immunization. In Kailali district of Nepal, health workers offered GMP and immunization on separate days.

Several health workers in Nepal and Ghana reported challenges with equipment for measuring weight (inadequate supply, infrequent replacement, and type of scale) and length (inadequate supply and difficulty transporting length/height boards to distant outreach clinics). A health worker from Jajarkot District, Nepal, noted:

*Sometimes when there are many children and when they cry*, *I face a little difficulty getting accurate measurements*. *Some children do not want to sit in weighing pants*. *We have a hanging type scale*, *but it would have been better if there was a scale for measuring the weight in [a] sitting position*.

A health worker from Garu District, Ghana, reported:

*The hanging scale[s] [don’t] keep long before they become faulty and start reading wrong measurements*.

### Growth promotion

During interviews in both Ghana and Nepal, health workers and caregivers showed a lack of clarity around “promotion,” especially in distinguishing it from growth monitoring. Despite the ambiguity, interviewees described and researchers observed key elements of promotion: 1) communicating results, 2) tailoring counseling, and 3) solving problems (including home visits and referral). As noted during observations and reported by government officials and health workers, availability of a growth trend, time, health worker workload, and skills influenced the quality of growth promotion in both countries.

#### Communicating results

During observed sessions, 75 percent (6 of 8) of health workers in Ghana communicated results of growth monitoring to caregivers and 67 percent (8 of 12) of health workers did so in Nepal. However, the information shared varied based on the information available from assessment and analysis. In Ghana, many health workers used the growth trend. In Nepal, some health workers used weight gain from the previous visit (three or more months ago) or, more commonly, whether the child’s current weight was in the normal range for their age.

#### Individual tailored counseling and problem solving

Several health workers in Ghana reported providing tailored counseling to caregivers after growth monitoring. Although the majority of health workers described tailoring counseling based on the growth trend, child’s age, and context, in practice, only two of the sessions we observed in Ghana were tailored (Table 4). During one of the two tailored sessions, the health worker asked the mother about the child’s diet and used the MCHRB to counsel the mother on the importance of a diverse diet. The mother actively participated by explaining the food she had been feeding the child and noting that the child had not been eating well. The health worker and caregiver then came to a specific agreement—the caregiver would try to increase the frequency of feeding and prepare the child’s food using a variety of food groups at home. Health workers and caregivers confirmed they covered topics such as breastfeeding, complementary feeding, and sometimes, developmental milestones during counseling. Health workers described listening to caregiver concerns, encouraging them for progress, and working with caregivers to solve problems. For example, one health worker in East Mamprusi described her approach:

*In counseling*, *you ask open-ended questions that will allow the mother to talk more rather [than] you doing the talking*. *This will help you explore the feeding practices of the mother and to know if [the] child is sick or not*. *These will help you identify the problem the caregiver has that is making her child not grow well*. *After identifying the feeding problem*, *you further engage the mother on options to address the problem until the two of you agree on what she can do to help address the problem*.

Despite demonstrated knowledge and skills described by health workers, we observed only 25 percent (2 of 8) of health workers in Ghana gave caregivers the opportunity to ask questions to work through problems together. Although health workers described informing caregivers when to return next month and recording it in the MCHRB, we did not observe health workers writing down agreed upon follow-up actions, which may have been due to time constraints. Nevertheless, mothers were confident they would be able to practice the agreed upon action (e.g., how to feed the child, where to go for a referral). A caregiver in East Mamprusi shared:

*I will be able to do what we agreed on for me to do…because I want the good health [for] my child and also the family will support me to do that*.

In contrast, because there was not an accurate growth trend, health workers and caregivers in Nepal described counseling tailored to whether the child was underweight. They focused only on children who were already malnourished and adjusted the information provided based on the child’s age. Children with growth faltering who were at risk of malnutrition were not identified and therefore not prioritized. Most health workers and FCHVs in Nepal did not describe receiving training on knowledge and skills specific to growth promotion.

When referring to counseling in Nepal, health workers, FCHVs, and caregivers frequently described unidirectional communication about age-based infant and young child feeding (IYCF) information. We observed the provision of information based on the child’s weight gain or weight-for-age (92 percent, 11 of 12). In contrast to caregiver descriptions of counseling, we observed that most health workers (67 percent, 8 of 12) attempted to involve the caregiver actively in the discussion and agreement. Health workers, including those from Kailali and Sindhupalchowk, respectively, reflected positively on caregiver engagement:

*Some [caregivers] come with [their] mother-in-law and husband; they show concern*. *They say they were suggested to feed particular foods and obeying the suggestion has done good for their children*.

*In the past*, *the community people didn’t listen to our suggestion of feeding nutritious food to their children*. *But*, *the situation has changed now*. *All are concerned about their child’s health*.

During observations in Nepal, we saw only one agreement between health worker and mother—on when to return—recorded. Although interviewed health workers described telling caregivers when to return, caregiver interviews and observations suggested the health worker proposed a timeframe based on when the child needed his or her next immunization rather than in a month for growth monitoring and promotion, per national guidelines. Overall, caregivers in Ghana and Nepal shared similar sentiments about the utility of GMP: during growth promotion, they learn new information about how to take care of their child and see their child improve after following instructions. One caregiver in Kailali noted:

*Yes*, *[GMP] was useful because we don’t know much about the [child’s] health*. *When health workers and FCHVs tell us*, *then we know how to take care of our children*.

#### Community mobilization and accountability

In Ghana, national and sub-national government officials and health workers emphasized the importance of community awareness about children’s growth and development and shared a variety of ways that the health system engaged community members. Examples included involving queen mothers (traditional female leaders) to support caregivers in making informed decisions around appropriate child feeding, sensitizing communities through radio talk shows, entertaining families at community *durbars*, and engaging fathers at home visits. A durbar is a formal community-wide gathering that includes cultural activities such as drumming and dancing; it provides an opportunity for sharing information with a large number of people simultaneously [23]. However, interview participants generally did not describe a mechanism for sharing GMP data with communities. Only one health worker described sharing GMP data with communities to encourage continued cooperation and district level feedback based on the data to stimulate collective action.

Similarly, in Nepal, systems to track growth-outcome data did not connect with efforts to stimulate collective action in community education by FCHVs. National and sub-national government officials described over-burdened local leadership, many of whom recently came into their roles after the government decentralized and had limited experience with GMP. Kailali was the only study district in Nepal where local leaders or *Bhalmansaa* supported community engagement, which may have contributed to higher attendance relative to Jajarkot and Sindhupalchowk, along with other factors. Some health workers in Nepal noted that FCHVs could take on a bigger role in community mobilization and in growth promotion activities. One sub-national government official from Nepal said:

*FCHV is an excellent model for reaching up to the community level and mothers groups*. *We have successfully increased growth monitoring indicators and improved nutrition status in the outreach centers with the help from FCHVs … FCHVs for conducting mothers’ group meetings have been very effective and this can be a model for other countries*.

#### Challenges

Several health workers in both contexts described heavy workloads due to staff attrition and competing priorities limited the amount of time they could spend with each caregiver during GMP sessions. In Ghana, high attendance also contributed to overloaded health workers. A national level government official and caregiver in Ghana, as well as several health workers in Nepal, shared that when time was limited, health workers cut short or skipped individualized counseling sessions. A government official from Nepal shared:

*Unlike one-to-one counseling*, *in group education*, *it is difficult to decipher caregivers who do not understand the counseling messages [and] some caregivers de-associate the information from their [unique] situation*. *When the crowd is large*, *especially on market days in rural areas (when transportation is available)*, *the health workers are simply unable to provide individualized counseling due to the numbers*.

A few caregivers and several health workers in Ghana noted that caregivers at health facilities and outreach points who need one-on-one counseling have to wait until health workers weigh all children. Several caregivers in Ghana and Nepal voiced a desire for more time with health workers related to child growth, development, and nurturing care. When we asked caregivers about other information they would like to learn during GMP, one caregiver in East Mamprusi said:

*If the nurses could also discuss with us how to talk*, *play*, *and interact with our children*, *it would help*, *because most of us don’t know how to do that*.

To overcome limited time with each caregiver, caregivers and health workers in Ghana reported that CHNs (with support from community health volunteers) conducted home visits for children who were growth faltering. These visits allowed them to problem solve based on the home context, follow up on recommendations, and engage family members. In contrast, in Nepal, due to the focus on whether a child was already underweight during GMP, most health workers and FCHVs could only conduct home visits with children who were malnourished, which they used to ensure completion of referrals.

### Data for decision-making

Interviewed health workers in both countries described systematically reporting growth monitoring data through the district health information system 2 (DHIS2) in Nepal and district health information management system (DHIMS) in Ghana. (DHIMS is built on DHIS2). For GMP, health workers uploaded weight-for-age data (normal weight, moderately underweight, severely underweight) disaggregated by age to a digital platform every month. In both countries, data entered into the digital platform were available for all levels of government to review data and ensure accuracy.

In Ghana, health workers reported use of growth monitoring data to identify useful group counseling topics, determine which geographic areas need special attention, assess whether GMP attendance (and other) targets were met, and lobby for additional resources. One sub-national government official from Garu District, Ghana, said:

*It helps in program planning in terms of logistics and human resources needed*. *For example*, *because of data we were able to convince the regional level to train all our staff in IYCF in Garu District*.

In Ghana, national and sub-national government officials described using GMP data to evaluate facility performance, identify areas for improvement, plan supportive supervision and nutrition interventions, and make funding decisions. However, some health workers and government officials reported that they did not use growth-monitoring data. Nongovernmental organizations and development partners receive growth-monitoring data for planning and programming.

In Nepal, several health workers mentioned using growth monitoring data to determine program reach, follow up on defaulters, and develop community-monitoring plans. However, several also mentioned that they did not use growth-monitoring data. At the sub-national level, government officials talked about using data to design programs, identify areas for supervision, and develop action plans. Several also noted that in the new decentralized federal structure, they did not play a decision-making role. Sub-national government officials from Jajarkot and Sindhupalchowk described conducting Routine Data Quality Audits (RDQA) in select health facilities every year to ensure accuracy of data reported in the electronic system. One sub-national government official from Nepal said:

*During the data quality audit*, *we check for the similarities and discrepancies between the data recorded in the registers*, *reports*, *and online database* … *We also find out the reasons behind the inconsistencies … After discussing with the health workers*, *we then conduct meetings with the local levels in presence of the mayor to discuss the problems seen during the data quality audit of health facilities and get their suggestions as well*.

Health workers did not report tracking data on growth promotion in a systematic way in either country. A review of the child health card and nutrition register revealed that health workers in Nepal record certain IYCF practices (e.g., exclusive breastfeeding) in the two tools. Similarly, upon reviewing the MCHRB and community-IYCF register, we learned that health workers in Ghana recorded IYCF practices, as well as whether they provided counseling.

## Discussion

This case study describes implementation of GMP in Ghana and Nepal to inform the global conversation initiated during the 2018 GMP convening. In this study, we examined growth monitoring, growth promotion, data use, and implementation challenges from the perspectives of several health system actors (national and sub-national government officials, health workers, and volunteers) and caregivers. We found that health workers in Ghana frequently plotted weight measurement and based promotion efforts on the growth trend for early detection of growth faltering and preventive actions. In contrast, health workers in Nepal did not always plot weight measurements and based promotion actions on weight from one point in time to identify children who were already underweight. Despite this difference, caregivers in both countries acknowledged the value of GMP and appreciated learning from health workers during GMP sessions. While both countries had a systematic way of tracking growth monitoring data, the way government officials and health workers used the data varied. Common implementation challenges were poor quality weighing scales, infrequent training, workload, and time. Our findings show that both GMP service delivery and demand need strengthening (Table 5).

**Table 5:** Summary of recommendations for successful GMP programs.

	Growth Monitoring	Growth Promotion
**Service Delivery**		
Develop global program guidance that covers…	How to assess and analyze the growth trend How to use data for program improvement and accountability	How to act based on the growth trend, age, and stage
Equip health worker with appropriate tools	Weighing scales that take accurate anthropometric measurements to assess growth in low resource settings Growth charts that enable visualization of an individual child’s growth curve for analysis	Growth charts that enable visualization of an individual child’s growth curve for action An algorithm to prioritize individual counseling based on the growth trend and child’s development
Train health workers on…	How to assess and analyze the growth trend How to use data for program improvement	How to act (e.g., referrals, counseling) based on the growth trend Mechanisms to task share such as by training community health volunteers on promotion
**Demand Generation**		
Engage household and community members through…	Promotion actions (e.g., home visits, community score card, child development) encourage attendance at GMP sessions to have frequent measurements for an accurate growth trend	Home visits for children who are growth faltering Community scorecards Integration of nurturing care during promotion actions
Provide caregivers tools to…	Visualize growth to encourage attendance at GMP sessions	Innovative visual tools for tailored counseling, group counseling, community mobilization, and community accountability

On the service delivery side, GMP programs would benefit from global guidance that covers appropriate design of the growth chart and how to assess, analyze, act, and use growth monitoring data. The guidance would support harmonization of GMP programs to align with best practices. Further, health workers need frequent and high quality capacity strengthening in the form of training and supervision for both growth monitoring and promotion [24]. Consistent with previous studies, our findings show the importance of health worker training for plotting weight on child health cards and quality counseling based on the growth trend [24,25]. Similarly, studies have also found that overburdened health workers have competing priorities, which leads to limited time with each caregiver to communicate growth results, provide tailored counseling, and problem solve [2,4,26,27].

Introducing new methods to task share with other cadres of health workers or volunteers and streamline workflow using a decision making algorithm would help address these constraints [28–34]. This would also allow health workers to have sufficient time to tailor counseling, solve problems, and reach agreements on actions to take. The algorithm could also help health workers prioritize home visits for children who are growth faltering or facing developmental delays while accounting for the home environment and engaging other family members [28,35–37]. Several studies show that caregivers of children who receive growth monitoring, individual nutrition counseling, and basic health care have better nutritional status and/or survival than those who only receive growth monitoring [4].

Data tracking and use is also critical for effective service delivery. In line with previous studies, we found few health workers use program data (growth monitoring in this case) from national health information systems for improving service delivery, leaving an opportunity for better utilization [38,39]. Additionally, health workers in this study did not have a place to enter growth promotion data into national health information monitoring systems. Governments will need to carefully consider how to best use promotion (including counseling) data before identifying indicators for tracking and entering into DHIS2.

Demand generation may be particularly important when distance from the health facility and competing demands make it difficult for caregivers to prioritize GMP [24], as was the case in Ghana and Nepal. Tools for visualizing growth and child development more broadly may encourage caregivers to prioritize GMP and attend more frequently which is necessary for accurate growth trends, early detection of growth faltering, and timely prevention measures [24,32]. Caregivers in both countries expressed a desire for more information on how their child is developing and how to play and interact with them. Previous studies have shown novel caregiving interventions around play and stimulation are of interest to caregivers and can contribute positively to program engagement [40–42]. At the community level, sharing and discussing aggregate GMP data through community score cards or boards may help community members encourage caregivers to attend GMP regularly and solve collective problems [28,43].

Further research is required on how to asses adequate growth (or growth faltering) based on weight. To date, programs have used several strategies (inspecting the growth curve, comparing it to the reference curve, minimum weight gain per month by age) to analyze growth faltering but there is no universally agreed upon approach [28,44]. Additionally, we found several challenges with the availability and maintenance of weighing scales in both countries. Innovative approaches to measure length are being tested [45]. We need similar innovative tools to measure weight in low resource settings. Finally, we need to better understand how to support caregivers to attend GMP service points since several factors influence their ability to bring their children for GMP.

## Limitations

This case study had several limitations. As with most qualitative research, purposive selection of respondents, small sample sizes, and the narrow scope of the research limit the generalizability of the findings beyond the time and place of research. In particular, in Ghana, both study districts were located in the north. Data collected were not necessarily representative of the entire district nor all GMP sessions in a community. As with all data collection, there was a risk of response bias, particularly from health workers or stakeholders potentially presenting their work in a favorable light. Study reliability and validity were also limited because we collected data on GMP services during the COVID-19 pandemic. Travel restrictions limited how extensively instruments could be pretested outside of the study areas and resulted in virtual rather than in-person interviews with national stakeholders.

## Conclusion

The findings of this study demonstrate that GMP programs may not always focus on the growth trend for early detection of growth faltering and preventive actions. Several factors contribute to these differences such as health worker training, time, and workload; design of growth charts; and availability of quality weighing scales. We recommend overcoming these challenges through actions to improve service delivery and generate demand for GMP. For improved service delivery, we suggest developing global guidance and ensuring health worker training and use of tools to assess, analyze, and act based on growth trends. For demand generation, we call for engaging household and community members through promotion actions, integrating early childhood development, and providing caregivers with tools to visualize growth and encourage attendance at GMP. Future research should document implementation learnings of these suggested actions in different contexts.

## Supporting information

S1 File Inclusivity in global research questionnaire. (DOCX)

S2 File Data from Ghana. (XLSX)

S3 File Data from Nepal. (XLSX)
