# Supplementary material for: Learning from health system actor and caregiver experiences in Ghana and Nepal to strengthen growth monitoring and promotion
Source: PLoS One. 2023 Mar 9;18(3):e0282807. doi: 10.1371/journal.pone.0282807 (PMC9997959; doi:10.1371/journal.pone.0282807)
Supplement: S1 File — (DOCX) [file pone.0282807.s001.docx]

Inclusivity in global research

PLOS’ policy on inclusivity in global research aims to improve transparency in the reporting of research performed outside of researchers’ own country or community and ensures that PLOS publications reporting global research adhere to high standards for research ethics and authorship. Authors of relevant research articles may be asked to complete the questionnaire below, which outlines ethical, cultural, and scientific considerations specific to inclusivity in global research. This questionnaire may be requested when researchers have travelled to a different country to conduct research, if research uses samples collected in another country, research with Indigenous populations or their lands, or if research is on cultural artefacts. Researchers travelling to another country solely to use laboratory equipment will not normally be required to complete the questionnaire. However, the questionnaire can be requested at the journal’s discretion for any submission – if you have been requested to complete this questionnaire by the PLOS journal you submitted to, please do so.

Please complete the questionnaire below and include this as a Supporting Information file with your manuscript. Note that if your paper is accepted for publication, this checklist will be published with your article in the supporting information files. Please ensure that you reference the checklist in the main body of your manuscript. We suggest adding a subsection ‘Inclusivity in global research’ to your Methods section and adding the following sentence: “Additional information regarding the ethical, cultural, and scientific considerations specific to inclusivity in global research is included in the Supporting Information (SX Checklist)”

The questions have been designed to be applicable to a wide range of study types, and there are subsections for both human subjects research and non-human subjects research. If any of the questions are not relevant to your research please mark them as “N/A” as appropriate.

**Ethical considerations, permits and authorship**

*This section is applicable to all research types.*

Provide details as to who granted permissions and/or consent for the study to take place in the Methods section of your manuscript. This should include the names of **all** ethics boards, governmental organizations, community leaders or other bodies that provided approval for the study. If individuals provided approval refer to these people by their role or title but do not list their name(s).

The study was reviewed and approved by John Snow Inc. Institutional Review Board (#20–34), the Ghana Health Service Ethics Review Committee (GHS-ERC013/10/20), and the Nepal Health Research Council (177/2021 P). All participants gave written (or thumb print) verbal consent before interviews and observations.

Reported on page number: 9

If there were any deviations from the study protocol after approval was obtained please provide details of these changes in the Methods section of your manuscript.
Did this study involve local collaborators that are residents of the country where the research was conducted or members of the community studied? If you do not have any authors from said communities, please provide an explanation for this below.

The study did involve local collaborators that are residents of the country where the research was conducted, Ghana and Nepal. They are co-authors on the manuscript.

There were no deviations from the study protocol after approval was obtained.

Reported on page number: N/A

Everyone listed as an author should meet PLOS’ criteria for authorship and all individuals who meet these criteria should be included in the author byline, rather than the acknowledgements. Authorship criteria is based on the International Committee of Medical Journal Editors (ICMJE) Uniform Requirements for Manuscripts Submitted to Biomedical Journals - for further information please see here: <https://journals.plos.org/plosone/s/authorship>.

**Human subjects research (e.g. health research, medical research, cross-cultural psychology)**

Did you obtain written informed consent from a representative of the local community or region before the research took place? How did you establish who speaks for the community? Details of written informed consent obtained from study participants should be reported separately in the Methods section of your manuscript.

How did members of the local community provide input on the aims of the research investigation, its methodology, and its anticipated outcome(s)?

Prior to data collection, in Ghana, we held a workshop with national stakeholders; and in Nepal, in collaboration with the Suaahara II project, we held three district-level workshops with key stakeholders. During these workshops we introduced stakeholders to the study and solicited feedback on the study plan/outcomes.

Ghana: In line with Ghana Health Service Ethics Review Committee procedures, we secured a letter from the Head of the Nutrition Department to Regional Health Directors (RHD) of the regions where the study was conducted to inform them of the study, share the schedule, and request their support. RHDs provided letters of support for ethical approval of the study, informed District Health Directors of the study districts, shared the list of selected health facilities, and requested they inform facilities. Regional Health Directorates also informed relevant regional staff (e.g., Regional Nutrition Officers and Regional Public Health Nurses) about the study.

Nepal: First, we coordinated with the USAID-funded Suaahara II project team for obtaining letters of support from the study districts. The support letters provided by the District Health Offices were submitted to the Nepal Health Research Council along with the study protocol. We then received an ethical clearance/ approval from the Nepal Health Research Council (NHRC). We submitted the NHRC's letter to all three local levels, one per district. The study commenced after receiving formal written approvals from the local levels/ municipalities. The health posts were requested to support the study by the local levels.

Written informed consent was obtained from all the study participants.

When engaging with the local community, how did you ensure that the informed consent documents and other materials could be understood by local stakeholders?

We aligned informed consent documents with preferred formats of local ethics review entities. We translated the documents to local languages as needed. Researchers took time to explain the study and what the participants were consenting to and answered any questions that came up.

Will the findings of the research be made available in an understandable format to stakeholders in the community where the study was conducted (e.g. via a presentation, summary report, copies of publications, etc.)? Please provide details of how this will be achieved.

Yes, the findings of the research have been made available in an understandable format to stakeholders in the community where the study was conducted. After the research was complete, we held workshops with stakeholders in Ghana and Nepal to share, contextualize, and validate key findings. We also produced short, easy-to-read narrative reports summarizing the study objectives, methodology, findings, lessons learned, and future opportunities which we shared back with key stakeholders. We also submitted these narratives as final reports to local IRBs and notified them of submission for publication.

**Non-human subjects research using specimens/ animals collected as part of the study, or those housed in archival collections. Examples include archaeology, paleontology, botany and zoology.**

Did the permission you obtained from a local authority to perform the study include an agreement on access to outputs and benefit sharing? This may include procedures to enable fair distribution of the benefits and resources arising from the research performed. Please include any details of Prior Informed Consent and Benefit Sharing Agreements obtained. These may be required by field-specific regulations, for example the Convention on Biological Diversity (CBD) and the associated Nagoya Protocol.

N/A

If the material used in your study was imported, please A) provide the year it was imported and B) indicate whether permits were obtained to import/export the materials used, C) provide details of any permits obtained. If this information is not available, please indicate this.

N/A

If you used archival specimens, please state how the material used in your study was acquired by the institute it is held in and provide details of any permits obtained for the original excavations/ sample collection. If this information is not available, please indicate this.

N/A

How was the potential cultural significance of the materials collected in your study to local communities considered in your research design? Were Indigenous peoples and/or local researchers and institutions involved with archaeological excavations / collection of specimens? If so, please provide a description of their involvement.

N/A

If your manuscript includes photographs of human remains please indicate whether authors obtained permission from descendants or affiliated cultural communities to do so.

N/A
